# Supplementary material for: Clinical Performance of Rapid and Point-of-Care Antigen Tests for SARS-CoV-2 Variants of Concern: A Living Systematic Review and Meta-Analysis
Source: Viruses. 2022 Jul 6;14(7):1479. doi: 10.3390/v14071479 (PMC9324571; doi:10.3390/v14071479)
Supplement: Supplementary file 1 [file viruses-14-01479-s001.zip › Text S1.pdf]

Text S1. Study protocol submitted to PROSPERO (registration: CRD42022329714).

PROSPERO STEP1

Will your registration record be in English?

YES NO

Is this a scoping, literature or mapping review?

YES NO

Does your review include a health outcome with direct relevance to human health?

(e.g. reviews of educational interventions to improve maths skills are not eligible, reviews of educational interventions to promote breastfeeding are eligible)

YES NO

Is this a Cochrane review?

YES NO

Is this a mini or partial review done for a training course or classwork or are you using the system to learn how to register?

PROSPERO does not have resources to process applications for reviews done only for training purposes. This includes mini reviews restricted to a subset of eligible studies, demonstrator reviews where a whole class does the same review, or any other projects that are less than full systematic reviews.

For learning purposes you may download and complete the PROSPERO form as a PDF document . If you do complete the form online, please save this in your own space and **do not SUBMIT it** for publication.

YES NO

Have you written a protocol?

PROSPERO registration captures key information about the design and conduct of a planned systematic review. It is not a full protocol. We strongly encourage you to write a full protocol before completing the PROSPERO registration form (although you may proceed without doing this).

YES NO

Will more than one person be involved in the systematic review?

We strongly recommend that you follow best practice and include more than one person in the review team. PROSPERO will not accept registrations unless there is more than one person conducting the review. You must include details of the other author(s) in the registration form.

YES NO

Do you intend to publish the results of your systematic review and/or make them publicly available when completed?

PROSPERO aims to increase transparency and help prevent unintended duplication of effort. This requires that the results of systematic reviews should be made publicly available e.g. by publication in an academic journal, posting in a research repository or being made available on a permanent website. We therefore do not accept registrations from systematic reviews that will not be made available to others e.g. projects that are internal to an organization or company, or masters dissertations if it is known that these will not be shared.

YES NO

Have you started your review?

YES NO

Stage of review

What work have you already done on your systematic review?

Preliminary searches

Not started Started Completed

Piloting the study selection process

Not started Started Completed

Formal screening of search results against eligibility criteria

Not started Started Completed

Data extraction

Not started Started Completed

Risk of bias (quality) assessment

Not started Started Completed

Data analysis

What stage is your review at regarding data analysis?

Not started Started Completed

Please now go ahead and register your review.

STEP 2 : PROSPERO 등록 (총 40개/ 필수★ 26개 항목)

1. Review title ★

SARS-CoV-2 rapid antigen test compared with RT-PCR assay for emerging variants: living systematic review

2. Original language title

코로나19 의심자에서 바이러스 변이형별 신속항원검사의 진단정확도: living 체계적 문헌고찰

3. Anticipated or actual start date ★

2022.02.01.

4. Anticipated completion date ★

2022.6.30.

5. Stage of review at time of this submission ★

[ ] The review has not yet started

**Review stage**	**Started**	**Completed**
Preliminary searches	[x]	[x]
Piloting of the study selection process	[x]	[x]
Formal screening of search results against eligibility criteria	[x]	[ ]
Data extraction	[ ]	[ ]
Risk of bias (quality) assessment	[ ]	[ ]
Data analysis	[ ]	[ ]

6. Named contact ★

Jimin Kim

7. Named contact email ★

jimin@neca.re.kr

8. Named contact address

Neungdong-ro, Gwangjin-gu, Seoul, Korea

9. Named contact phone number

82221742766

10. Organisational affilation of the review ★

National Evidence-based Healthcare Collaborating Agency

11. Review team members and their organisational affiliations ★

Jimin Kim / NECA / jimin@neca.re.kr

Seri Jeong/ Kangnam Sacred Heart Hospital, Hallym University College of Medicine/hehebox73@hallym.or.kr

12. Funding sources/sponsors ★

National Evidence-based Collaborating Agency, Republic of Korea [grant number NECA-A-22-009]

13. Conflicts of interest ★

None

14. Collaborators

None

15. Review question ★

To evaluate the performance of the COVID-19 rapid antigen test as an AgPOCT for the diagnosis of SARS-CoV-2 infection in nasopharyngeal samples compared with an RT-PCR assay.

- Population: A person suspected of COVID-19
- Index test: Rapid antigen test (visually readable equipment)
- Reference test: RT-PCR
- Outcome: Test accuracy (sensitivity, specificity)

16. Searches ★

We systematically searched Ovid-MEDLINE, OvidEMBASE, and CENTRAL, as well as the Korean databases (KMBASE) through April 10th 2022. We did not limit the publication language and there was limitation to year of publication starting from 2020.

17. URL to search strategy

None

18. Condition or domain being studied ★

Identifying COVID-19 infected patients

19. participants/population ★

We will include studies with any population (adults and children, symptomatic and asymptomatic) and in any setting for COVID-19. Participants came to the site for Covid-19 testing because they had symptoms or were asymptomatic but seeking testing for possible Covid-19 exposure or other reasons.

20. intervention, exposure ★

Point-of-care testing uses rapid diagnostic tests performed or interpreted by someone other than the individual being tested or their parent or guardian and can be performed in a variety of settings. Results for the antigen test were read on site and classified as positive, negative, inconclusive. A study was performed using nasopharyngeal or nasal swabs.

21. Comparator/control ★

A SARS-CoV-2 RT-PCR was considered as the gold-standard.

22. Types of study to be included ★

We will consider randomized clinical trials (RCTs) or observational (e.g. cohort, case-control) quantitative studies that produce estimates of test accuracy or provide data from which we can compute estimates.

23. Context

About the Ct value, and 30 or more contains 10% or more of the sample were included according to The KFDA standards.

We will include studies that provide the 2x2 table (number of true positives [TP], false negatives [FN], true negatives [TN] and false positives [FP]) or joint classification-tables of index tests.

24. Main outcome ★

The main outcome is to assess sensitivity and specificity of rapid antigen for screening for COVID-19.

25. Additional outcome ★

The significant difference is that PPV and NPV use the prevalence of a condition to determine the likelihood of a test diagnosing that specific disease.

26. Data extraction (selection and coding) ★

Data were extracted from the text, tables, and figures in the articles. A standardised, pre-piloted form will be used to extract data from the included studies for assessment of study quality and evidence synthesis. Two review authors extracted information from each included trial. These evaluations were carried out independently and yielded separate assessments. The disagreement was resolved by discussion and third opinion. The following information was included on the data extraction form: first author, publication date, study design, number of study

subjects, name of tool kit, and TP, FN FP, TN.

27. Risk of bias (quality) assessment ★

Two authors independently assessed the quality of the selected studies using the QUADAS-2 tool. Patient selection, index test, reference standard and flow and timing are the four core domains of the QUADAS-2 tool. The risk of bias was classified as low, high or unclear for each domain. Disagreements were addressed by consensus with the participation of a third review author.

28. Strategy for data synthesis ★

For the diagnostic accuracy of each index test, we will use the 2x2 table from individual studies as defined by the results of the index test against the reference standard. For each index test, a random-effects DTA meta-analysis will be performed. We will use the bivariate model to estimate a summary sensitivity and specificity with associated 95% confidence intervals (CIs), and positive and negative likelihood ratios of each index test.

29. analysis of subgroups or subsets ★

We will conduct subgroup analyses by variants of COVID-19, asymptomatic vs. symptomatic, adults vs. child, prevalence, enrollment periods.

30. type and method of review (choice) ★

- type of review: diagnostic Meta-analysis, living systematic review, systematic review
- health area of the review: Infectious and infestations Disease, COVID-19, diagnosis

31. Language

English, Korean

32. Country ★

South Korea

33. Other registration details

34. reference and/or URL for published protocol

35. dissemination plans

36. keywords

COVID-19, SARS-CoV-2, Point of care, Rapid antigen test

37. details of any existing review of the same topic by the same authors

none

38. current review status★

ongoing

39. any additional information

40. details of final report/publications(s) or preprints if available
