# Supplementary material for: Clinical Performance of Rapid and Point-of-Care Antigen Tests for SARS-CoV-2 Variants of Concern: A Living Systematic Review and Meta-Analysis
Source: Viruses. 2022 Jul 6;14(7):1479. doi: 10.3390/v14071479 (PMC9324571; doi:10.3390/v14071479)
Supplement: Supplementary file 1 [file viruses-14-01479-s001.zip › Text S2.pdf]

Text S2. Search formula
(PubMed Search; adapted for other searches)

PubMed (2021.06.29.)

No.	Query	Items found
#1	"COVID-19"[Mesh]	88,128
#2	"COVID-19"[TW] OR "COVID 19"[TW] OR "COVID-19 Virus Disease"[TW] OR "COVID 19 Virus Disease"[TW] OR "COVID-19 Virus Diseases"[TW] OR "Disease, COVID-19 Virus"[TW] OR "Virus Disease, COVID-19"[TW] OR "COVID-19 Virus Infection"[TW] OR "COVID 19 Virus Infection"[TW] OR "COVID-19 Virus Infections"[TW] OR "Infection, COVID-19 Virus"[TW] OR "Virus Infection, COVID-19"[TW] OR "2019-nCoV Infection"[TW] OR "2019 nCoV Infection"[TW] OR "2019-nCoV Infections"[TW] OR "Infection, 2019-nCoV"[TW] OR "Coronavirus Disease-19"[TW] OR "Coronavirus Disease 19"[TW] OR "2019-nCoV Disease"[TW] OR "2019 nCoV Disease"[TW] OR "2019-nCoV Diseases"[TW] OR "Disease, 2019-nCoV"[TW] OR "COVID19"[TW] OR "Coronavirus Disease 2019"[TW] OR "Disease 2019, Coronavirus"[TW] OR "SARS Coronavirus 2 Infection"[TW] OR "SARS-CoV-2 Infection"[TW] OR "Infection, SARS-CoV-2"[TW] OR "SARS CoV 2 Infection"[TW] OR "SARS-CoV-2 Infections"[TW] OR "COVID-19 Pandemic"[TW] OR "COVID 19 Pandemic"[TW] OR "COVID-19 Pandemics"[TW] OR "Pandemic, COVID-19"[TW] OR "2019 Novel Coronavirus Disease"[TW] OR "2019 Novel Coronavirus Infection"[TW] OR "coronavirus disease 2"[TW] OR "coronavirus disease 2019 pneumonia"[TW] OR "coronavirus infection 2019"[TW] OR "COVID"[TW] OR "COVID 19 induced pneumonia"[TW] OR "COVID 2019"[TW] OR "COVID-19 induced pneumonia"[TW] OR "COVID-19 pneumonia"[TW] OR "nCoV 2019 disease"[TW] OR "nCoV 2019 infection"[TW] OR "paucisymptomatic coronavirus disease 2019"[TW] OR "SARS coronavirus 2 pneumonia"[TW] OR "SARSCoV2 disease"[TW] OR "SARS-CoV2 disease"[TW] OR "SARS-CoV-2 disease"[TW] OR "SARSCoV2 infection"[TW] OR "SARS-CoV2 infection"[TW] OR "SARS-CoV-2 pneumonia"[TW] OR "severe acute respiratory syndrome 2"[TW] OR "severe acute respiratory syndrome 2 pneumonia"[TW] OR "severe acute respiratory syndrome coronavirus 2 infection"[TW] OR "severe acute respiratory syndrome coronavirus 2019 infection"[TW] OR "severe acute respiratory syndrome CoV-2 infection"[TW] OR "Wuhan coronavirus disease"[TW] OR "Wuhan coronavirus infection"[TW] OR "2019 novel coronavirus epidemic"[TW] OR "new coronavirus pneumonia"[TW] OR "novel coronavirus 2019 disease"[TW] OR "novel coronavirus 2019 infection"[TW] OR "novel coronavirus disease 2019"[TW] OR "novel coronavirus infected pneumonia"[TW] OR "novel coronavirus infection 2019"[TW] OR "novel coronavirus pneumonia"[TW] OR "2019nCoV"[TW] OR "19nCoV"[TW] OR "COVID19*"[TW] OR "SARSCOV-2"[TW] OR "SARSCOV2"[TW] OR "corona virus 2"[TW] OR "Wuhan"[TW] OR "Hubei"[TW] OR "new coronavirus"[TW] OR "novel coronavirus"[TW] OR "novel corona virus"[TW] OR "novel CoV"[TW]	152,152
#3	"SARS-CoV-2"[Mesh]	68,383

#4	"SARS-CoV-2"[TW] OR "Coronavirus Disease 2019 Virus"[TW] OR "Wuhan Seafood Market Pneumonia Virus"[TW] OR "SARS-CoV-2 Virus"[TW] OR "SARS CoV 2 Virus"[TW] OR "SARS-CoV-2 Viruses"[TW] OR "Virus, SARS-CoV-2"[TW] OR "2019-nCoV"[TW] OR "COVID-19 Virus"[TW] OR "COVID 19 Virus"[TW] OR "COVID-19 Viruses"[TW] OR "Virus, COVID-19"[TW] OR "Wuhan Coronavirus"[TW] OR "Coronavirus, Wuhan"[TW] OR "SARS Coronavirus 2"[TW] OR "Coronavirus 2, SARS"[TW] OR "Severe Acute Respiratory Syndrome Coronavirus 2"[TW] OR "2019 Novel Coronavirus"[TW] OR "2019 Novel Coronaviruses"[TW] OR "Coronavirus, 2019 Novel"[TW] OR "Novel Coronavirus, 2019"[TW] OR "2019 nCOV"[TW] OR "2019 severe acute respiratory syndrome coronavirus 2"[TW] OR "HCoV-19"[TW] OR "Human coronavirus 2019"[TW] OR "nCoV-2019"[TW] OR "SARS2 (virus)"[TW] OR "SARS-related coronavirus 2"[TW] OR "Sever acute respiratory syndrome coronavirus 2"[TW] OR "Severe acute respiratory coronavirus 2"[TW] OR "Severe acute respiratory syndorme coronavirus 2"[TW] OR "severe acute respiratory syndrome 2 virus"[TW] OR "severe acute respiratory syndrome corona virus 2"[TW] OR "severe acute respiratory syndrome coronavirus 2019"[TW] OR "Severe acute respiratory syndrome coronoavirus 2"[TW] OR "Severe acute respiratory syndrome coronvirus 2"[TW] OR "severe acute respiratory syndrome CoV-2 virus"[TW] OR "Severe acute respiratory syndrome related coronavirus 2"[TW] OR "Severe acute respiratory syndrome virus 2"[TW] OR "Severe acute respiratoy syndrome coronavirus 2"[TW] OR "2019 new coronavirus"[TW] OR "novel 2019 coronavirus"[TW] OR "novel coronavirus-19"[TW]	94,004
**#5**	**#1 OR #2 OR #3 OR #4**	**152,559**
#6	"Point-of-Care Testing"[Mesh]	2,746
#7	"Point-of-Care Testing"[TW] OR "Testing, Point-of-Care"[TW] OR "Point-Of-Care Diagnostic Testing"[TW] OR "Diagnostic Testing, Point-Of-Care"[TW] OR "Point Of Care Diagnostic Testing"[TW] OR "Testing, Point-Of-Care Diagnostic"[TW] OR "Point-Of-Care Diagnostic Tests"[TW] OR "Diagnostic Test, Point-Of-Care"[TW] OR "Diagnostic Tests, Point-Of-Care"[TW] OR "Point Of Care Diagnostic Tests"[TW] OR "Point-Of-Care Diagnostic Test"[TW] OR "Test, Point-Of-Care Diagnostic"[TW] OR "Tests, Point-Of-Care Diagnostic"[TW] OR "Point-Of-Care Tests"[TW] OR "Point Of Care Tests"[TW] OR "Point-Of-Care Test"[TW] OR "Point Of Care Test"[TW] OR "Test, Point-Of-Care"[TW] OR "Tests, Point-Of-Care"[TW] OR "Point of Care Testing"[TW] OR "Point-Of-Care Diagnostics"[TW] OR "Diagnostic, Point-Of-Care"[TW] OR "Diagnostics, Point-Of-Care"[TW] OR "Point Of Care Diagnostics"[TW] OR "Point-Of-Care Diagnostic"[TW] OR "Bedside Testing"[TW] OR "Testing, Bedside"[TW] OR "bed-side testing"[TW] OR "POCT"[TW] OR "POC"[TW] OR "POC test"[TW] OR "POC testing"[TW] OR Point Of Care test*[TW] OR "point-of-care antigen"[TW] OR "rapid antigen test"[TW] OR "rapid molecular test"[TW] OR "rapid test"[TW] OR "rapid testing"[TW] OR "rapid antigen"[TW]	19,161
**#8**	**#6 OR #7**	**19,208**
**#9**	**#5 AND #8**	**1,002**
**#10**	**#9 AND (2020/1/1:2021/12/31[pdat])**	**1,002**
